# The oxidoreductase activity of Rnf balances redox cofactors during fermentation of glucose to propionate in *Prevotella*

**DOI:** 10.1038/s41598-023-43282-9

**Published:** 2023-09-30

**Authors:** Bo Zhang, Christopher Lingga, Hannah De Groot, Timothy J. Hackmann

**Affiliations:** Department of Animal Science, University of California, Davis, CA USA

**Keywords:** Bacterial genomics, Bacterial physiology

## Abstract

Propionate is a microbial metabolite formed in the gastrointestinal tract, and it affects host physiology as a source of energy and signaling molecule. Despite the importance of propionate, the biochemical pathways responsible for its formation are not clear in all microbes. For the succinate pathway used during fermentation, a key enzyme appears to be missing—one that oxidizes ferredoxin and reduces NAD. Here we show that Rnf [ferredoxin—NAD^+^ oxidoreductase (Na^+^-transporting)] is this key enzyme in two abundant bacteria of the rumen (*Prevotella brevis* and *Prevotella ruminicola*). We found these bacteria form propionate, succinate, and acetate with the classic succinate pathway. Without ferredoxin:NAD^+^ oxidoreductase, redox cofactors would be unbalanced; it would produce almost equal excess amounts of reduced ferredoxin and oxidized NAD. By combining growth experiments, genomics, proteomics, and enzyme assays, we point to the possibility that these bacteria solve this problem by oxidizing ferredoxin and reducing NAD with Rnf [ferredoxin—NAD^+^ oxidoreductase (Na^+^-transporting)]. Genomic and phenotypic data suggest many bacteria may use Rnf similarly. This work shows the ferredoxin:NAD^+^ oxidoreductase activity of Rnf is important to propionate formation in *Prevotella* species and other bacteria from the environment, and it provides fundamental knowledge for manipulating fermentative propionate production.

## Introduction

Metabolites formed via anaerobic fermentation in the gastrointestinal of mammals have great effects on the host physiology and health^1,2^. The major metabolites formed by gut bacteria during fermentation of dietary carbohydrates are short-chain fatty acids (SCFAs). As one of the major SCFAs, propionate can affect satiety and glucose homeostasis in humans^3,4^. It has beneficial effects on beta-cell function to maintain healthy glucose homeostasis in humans^5^. Recently, it has been demonstrated that propionate can suppress colorectal cancer growth^6,7^, while excess levels of propionate may lead to Alzheimer’s disease, such as by inducing hyperammonemia^8^. Furthermore, propionate also plays important roles in other animals, such as ruminants. It is a major source of glucose for the ruminants, and about 50% of glucose is from propionate^9^. Propionate formation in the rumen of ruminants is negatively related with methane emission, since they compete for metabolic hydrogen in the rumen. Favoring propionate formation could mitigate methane emission^10^. Realizing its importance in human health, agricultural production, and the environment, studies focusing on biochemical pathways have revealed many enzymes responsible for fermentative propionate production^11,12^.

Three biochemical pathways are responsible for fermentative propionate production from dietary carbohydrates, including the succinate pathway, the acrylate pathway, and the propanediol pathway^11,12^. Propionate is most commonly formed using the succinate pathway and in combination with acetate. This pathway involves the conversion of succinate to propionate via methylmalonyl-CoA. Such organisms with this pathway include *Bacteroides fragilis*^13^ and *Selenomonas ruminantium*^14^. The acrylate pathway involves the conversion of lactate to lactoyl-CoA, acryloyl-CoA, propionyl-CoA and propionate, e.g. *Coprococcus catus*^11^ and *Megasphaera elsdenii*^15^. Some gut commensal bacteria, such as *Roseburia inulinivorans*^16^, carry out the propanediol pathway to form propionate from deoxy sugars. 

Despite decades of study, one major biochemical pathway (the succinate pathway) for forming propionate has unknown steps. When glucose is the substrate, this pathway has a problem: it forms excess amounts of reduced ferredoxin (Fd_red_), a redox cofactor (Supplementary Fig. S1A)^17^. This cofactor is formed by the enzyme pyruvate:ferredoxin oxidoreductase (EC 1.2.7.1), and no step is known to oxidize it back to ferredoxin (Fd_ox_) during this pathway. Similarly, the pathway forms oxidized NAD (NAD_ox_), with no step to reduce it back to reduced NAD (NAD_red_). This is an apparent problem in both prokaryotes^17,18^ and eukaryotes^19^. These unknown steps are significant because if Fd_ox_ and NAD_red_ are exhausted (not regenerated), fermentation will halt. The general importance of balancing Fd_ox_/Fd_red_ and NAD_ox_/NAD_red_ in fermentation has been recognized for decades^20,21^.

We hypothesized that the enzyme Rnf [ferredoxin—NAD^+^ oxidoreductase (Na^+^-transporting), EC 7.2.1.2] fills in the missing steps (Supplementary Fig. S1B) by simultaneously oxidizing Fd_red_ and reducing NAD_ox_, solving two problems at once. This enzyme plays a similar role in other pathways, such as one metabolizing caffeate^22^. Recently, we found Rnf genes in many propionate-forming bacteria from the rumen^17^. Here we study two of these rumen bacteria in detail and find that they indeed need the ferredoxin:NAD^+^ oxidoreductase activity of Rnf in forming propionate (or its precursor, succinate). We show this using growth experiments, genomics, proteomics, and enzyme assays, although genetic studies of *rnf* genes in these *Prevotella* species are missing. Further, we find Rnf is common in bacteria that form propionate (or succinate), with 39 type strains from many habitats encoding it. This work suggests the ferredoxin:NAD^+^ oxidoreductase activity of Rnf is important to propionate formation in many bacteria from the environment.

## Results

### Prevotella form propionate, succinate, and acetate during fermentation

Our hypothesis was that fermentation of glucose to propionate, succinate, and acetate uses the ferredoxin:NAD^+^ oxidoreductase activity of Rnf. Two bacteria isolated from the rumen (*Prevotella brevis* GA33 and *Prevotella ruminicola* 23) were used to test this hypothesis. We tested if they form propionate, succinate, and acetate and if these products are in the ratios as expected in Supplementary Fig. S1. We grew these bacteria on media containing glucose and ammonia, then analyzed the culture for several products. For *P*. *brevis* GA33, we used a medium that also contained yeast extract and trypticase, as it would not grow on media with glucose only. 

**Figure 1:**
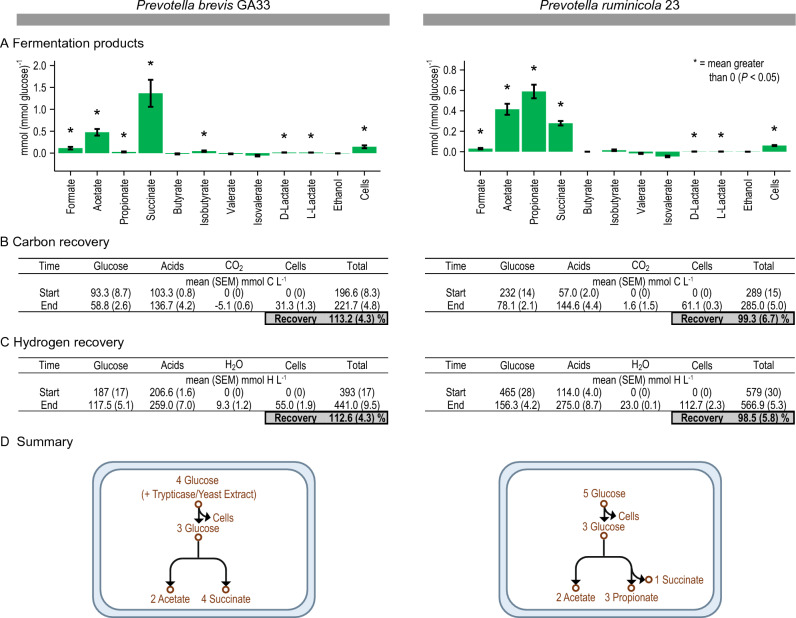
*Prevotella* form propionate, succinate, and acetate during fermentation of glucose. (**A**) Yield of fermentation products. (**B**) Recovery of carbon is near or above 100%. (**C**) Recovery of hydrogen is also near or above 100%. (**D**) Summary of growth and fermentation. In (A), the yield of cells is g (mmol glucose)^−1^. Results are mean ± standard error of at least 3 biological replicates (culture supernatant or cells prepared from independent cultures).

Both species formed large amounts of succinate and acetate (Fig. 1A). Propionate was formed in large amounts by *P*. *ruminicola* 23, whereas it was formed in only trace amounts by *P*. *brevis* GA33 (Supplementary Fig. S2). The ratio of succinate plus propionate to acetate was approximately 2:1. They also formed formate, D-lactate, and L-lactate, but only in trace amounts. These results follow our expectations (Supplementary Fig. S1A).

Neither species formed H_2_ (Supplementary Fig. S3). As a control, we analyzed gas samples from *S. ruminantium* HD4, a propionate-forming bacterium that forms H_2_ in trace amounts^23^. We were indeed able to detect H_2_ formation by this organism (Supplementary Fig. S3). This result shows that we would have been able to detect H_2_ if these *Prevotella* species formed it, even in trace amounts.

To check how accurately we measured these products, we calculated carbon and hydrogen recovery (Fig. 1B,C). A recovery of 100% indicates that all carbon (or hydrogen) at the start of incubation was recovered in products measured at the end. We found recoveries were near or above 100%. For *P*. *brevis* GA33, values were above 100% because our calculations did not account for trypticase and yeast extract in the medium of this bacterium. The high recoveries of carbon and hydrogen indicate that we measured all products accurately.

In sum, our work shows that *Prevotella* species form propionate, succinate, and acetate as the sole products of fermentation (Fig. 1D). Additionally, they form these products in the ratio expected (Supplementary Fig. S1).

### Redox cofactors in Prevotella species appear to be unbalanced

We hypothesized that both *Prevotella* species use the ferredoxin:NAD^+^ oxidoreductase activity of Rnf to balance fermentation. Without this enzyme, fermentation should produce excess NAD_ox_ and Fd_red_. We determined if this was indeed the case for these *Prevotella* species. 

We calculated the quantity of NAD_ox_ and Fd_red_ produced during the experiments above (Supplementary Table S1). Our calculations revealed that excess NAD_ox_ and Fd_red_ were indeed formed. The amount was 2.7 NAD_ox_ and 2.3 Fd_red_ per 3 glucose (Supplementary Table S1). This was even higher than expected (Supplementary Fig. S1) and owed to additional NAD_ox_ and Fd_red_ being formed during production of cells (particularly lipid) (Supplementary Table S2). This calculation did not include ferredoxin:NAD^+^ oxidoreductase activity, and it shows fermentation would indeed be unbalanced without this enzyme.

Next, we calculated how long cells could sustain unbalanced redox cofactors during fermentation. The calculation showed that all Fd_ox_ would be consumed and fermentation would halt within 1.5 s. This calculation assumes 2.3 Fd_red_ per 3 glucose fermented (Supplementary Table S1), 667 nmol glucose fermented (g dry cells)^−1^ s^−1^ (see Methods), 75 nmol total ferredoxin/g wet cells^24^, wet cells are 10% dry mass, and all ferredoxin starts as Fd_ox_. Without the ferredoxin:NAD^+^ oxidoreductase or other enzyme(s) to regenerate NAD_red_ and Fd_ox_, cells could sustain fermentation only for seconds (or less).

We performed calculations on *P. ruminicola* 23 only. To calculate the quantity of NAD_ox_ and Fd_red_ formed during production of cells, we assumed macromolecules were synthesized from glucose and ammonia (Supplementary Table S2). This would have been a bad assumption for *P. brevis* GA33, where macromolecules could have come from trypticase and yeast extract.

Our calculation points to an apparent excess of NAD_ox_ and Fd_red_ formed during fermentation and growth. It shows a critical need for ferredoxin:NAD^+^ oxidoreductase activity of Rnf or other enzyme(s) that can regenerate NAD_red_ and Fd_ox_.

### Prevotella have Rnf [ferredoxin—NAD^+^ oxidoreductase (Na^+^-transporting)]

Having established a need for an enzyme like Rnf, we determined if this activity of Rnf is indeed possessed by these *Prevotella* species. Genomics, proteomics, and enzyme assays were used to test its presence.

**Figure 2:**
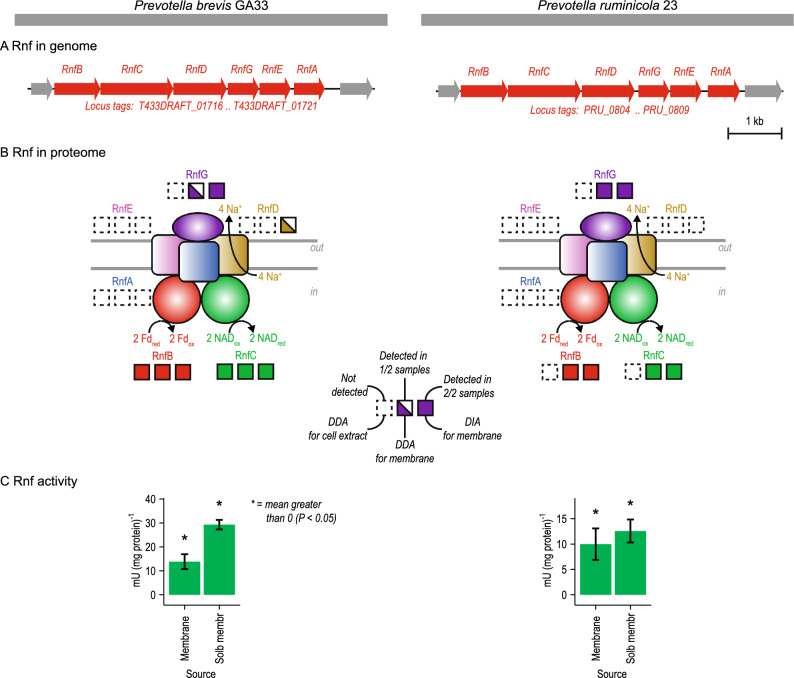
*Prevotella* have the enzyme Rnf**.** Rnf is evident in the (**A**) genome, (**B**) proteome, and (**C**) measurements of enzyme activity. Figures shown on the left side are for *Prevotella brevis* GA33, while figures shown on the right side are for *Prevotella ruminicola* 23. Results in (**C**) are mean ± standard error of 3 biological replicates (cell membranes prepared from independent cultures). Abbreviations: Fd_ox_, oxidized ferredoxin; Fd_red_, reduced ferredoxin (two reduced iron-sulfur clusters); NAD_ox_, oxidized NAD; NAD_red_, reduced NAD; DDA, data-dependent acquisition; DIA, data-independent acquisition; Membrane, cell membrane sample; Solb membr, solubilized cell membrane sample. See Supplementary Tables S3 and S4 for more information.

*rnf* genes were identified in the genome and Rnf was found in the proteome (Fig. 2 and Supplementary Tables S3–S8). The genomes of both species had genes for all six subunits of this enzyme (Fig. 2A). Proteomics revealed genes for four subunits were expressed in *P*. *brevis* GA33 and three in *P*. *ruminicola* 23 (Fig. 2B). We used multiple sample types (cell extract, cell membrane) and applied different data-acquisition methods (data-dependent acquisition, data-independent acquisition) for the proteomic analysis. The two subunits we never detected (RnfA and RnfE) are integral proteins^25^, which may explain why it is challenging to detect them. RnfE has evaded detection even in purified Rnf^26^.

After finding evidence of Rnf in the genome and proteome, we tested for its catalytic activity [ferredoxin:NAD^+^ oxidoreductase] with enzyme assays (Fig. 2C). To do so, we measured formation of NAD_red_ by cell membranes after adding Fd_red_. We found that cell membrane of both *Prevotella* species had activity (Fig. 2C). The activity depended on adding both Fd_red_ and NAD_ox_. Further, the activity was localized to the membrane, whereas the activity in cytoplasmic contents was low for *P*. *brevis* GA33, 3.1 ± 0.5 (mean ± standard error of mean) mU/mg, and undetectable for *P. ruminicola* 23. When we performed a partial purification of Rnf (by solubilizing cell membranes in detergent), we found higher activity of this enzyme (Fig. 2C). These experiments show both *Prevotella* species had ferredoxin:NAD^+^ oxidoreductase activity of Rnf, and the properties were as expected. Likewise, these experiments rule out the presence of a similar enzyme in the cytoplasm [a cytoplasmic ferredoxin:NAD^+^ oxidoreductase].

**Figure 3:**
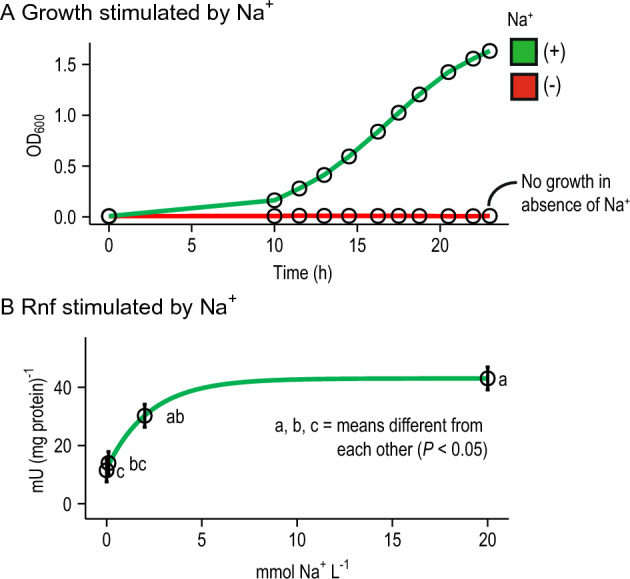
In *Prevotella brevis* GA33, growth and Rnf activity are stimulated by Na^+^. In (**A**), sodium ions were removed from the media by replacing NaCl, NaOH and Na_2_CO_3_ with equimolar KCl, KOH, and K_2_CO_3_. Resazurin was also removed. Results are for one representative culture. Experiments were done with 2 cultures on 2 different days. In (**B**), sodium ions were removed from the assay mix by replacing NAD sodium salt, sodium pyruvate, and CoA lithium salt with equimolar NAD hydrate, potassium pyruvate, and CoA hydrate. The residual Na^+^ in the Tris–Cl buffer and MgCl_2_ was 2 µM (as measured by an electrode; Fisher Accumet 13–620-503A). Results are mean ± standard error of 4 biological replicates (cell membranes prepared from independent cultures).

To verify that this activity was due to Rnf but not another enzyme (e.g. possible alternatives to Rnf, see below), we determined if the activity was stimulated by sodium ions (Fig. 3). In most species, Rnf pumps sodium ions (to create a gradient) and thus depends on them for high activity^26–28^. We found that *P*. *brevis* GA33 did not grow without sodium ions, showing a general dependence on sodium ions (Fig. 3A). This was the same for *P*. *ruminicola* 23 (data not shown). In addition, sodium ions directly stimulated the ferredoxin:NAD^+^ oxidoreductase activity of Rnf (Fig. 3B). We attempted to show pumping of sodium ions directly with inverted membrane vesicles, but attempts at creating vesicles failed.

Our enzyme assays required Fd_red_, which we generated using a system similar to Schoelmerich et al.^29^. Specifically, we purified ferredoxin from *Clostridium* *pasteurianum* 5, then we reduced it with pyruvate and crude pyruvate:ferredoxin oxidoreductase. The crude pyruvate:ferredoxin oxidoreductase was the cytoplasmic contents prepared from the organism in which Rnf was tested (*P. brevis* GA33 or *P. ruminicola* 23). We verified that the crude pyruvate:ferredoxin oxidoreductase worked as intended. First, we used it to detect activity of Rnf in *Pseudobutyrivibrio ruminis* A12-1. We found activity of 50.0 ± 1.9 mU/mg, which is similar to the value found by Schoelmerich et al.^29^ for the same organism. Second, we screened it for activity of interfering enzymes, including a cytoplasmic ferredoxin:NAD^+^ oxidoreductase and pyruvate dehydrogenase. We found these interfering activities were low or undetectable [see results above for cytoplasmic ferredoxin:NAD^+^ oxidoreductase and see below for pyruvate dehydrogenase]. These results show this system is appropriate for generating Fd_red_ in the assays for measuring ferredoxin:NAD^+^ oxidoreductase activity.

In sum, our work establishes that these *Prevotella* species have Rnf as well as the ferredoxin:NAD^+^ oxidoreductase activity of Rnf at the genomic, proteomic, and enzymatic level. With it, these *Prevotella* species can handle excess NAD_ox_ and Fd_red_ produced during fermentation.

### Prevotella have other enzymes needed to form fermentation products

**Figure 4:**
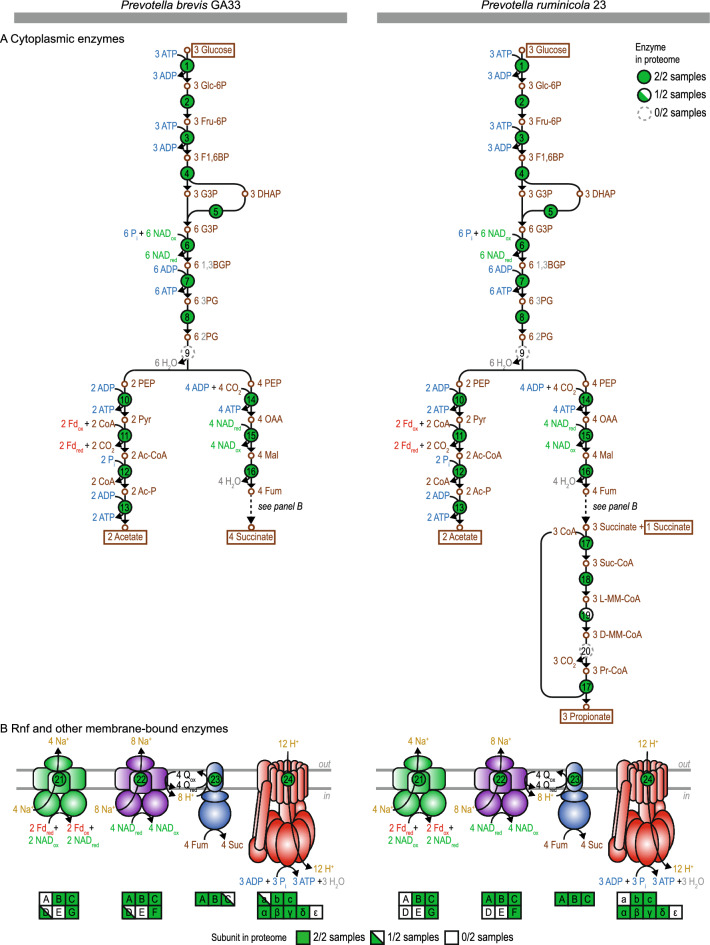
*Prevotella* have enzymes for forming propionate, succinate, and acetate in the proteome**.** (**A**) Cytoplasmic enzymes. (**B**) Rnf and other membrane-bound enzymes. Abbreviations: Glc-6P, glucose-6-phosphate; Fru-6P, fructose-6-phosphate; F1,6BP, fructose-1,6-bisphosphate; G3P, glyceraldehyde-3-phosphate; DHAP, dihydroxyacetone phosphate; 1,3BGP, 1,3-bisphosphoglycerate; 3PG, 3-phosphoglycerate; 2PG, 2-phosphoglycerate; PEP, phosphoenolpyruvate; Pyr, pyruvate; Ac-CoA, acetyl-CoA; Ac-P, acetyl-phosphate; OAA, oxaloacetate; Mal, malate; Fum, fumarate; Suc-CoA, succinyl-CoA; L-MM-CoA, L-methylmalonyl-CoA; D-MM-CoA, D-methylmalonyl-CoA; Pr-CoA, propionyl-CoA; Fd_ox_, oxidized ferredoxin; Fd_red_, reduced ferredoxin (two reduced iron-sulfur clusters); NAD_ox_, oxidized NAD; NAD_red_, reduced NAD; CoA, coenzyme A; P_i_, inorganic phosphate; Q_ox_, oxidized quinone; Q_red_, reduced quinone. See Supplementary Tables S3 and S4 for more information.

After finding that Rnf was present in both *Prevotella* species but not those possible alternatives to Rnf, we determined if other enzymes forming propionate, succinate, and acetate were also present using the combination of genomics, proteomics, and enzyme assays. This was important to confirm that redox cofactors (NAD_ox_ and Fd_red_) are produced in the pathway as expected. We found enzymes of the classic succinate pathway in the genome and proteome (Fig. 4 and Supplementary Tables S3–S8). When using proteomics, we found cytoplasmic enzymes for glucose fermentation were well detected (Fig. 4A). Membrane-bound proteins (e.g. Nqr [EC 7.2.1.1], fumarate reductase [EC 1.3.5.1], and ATP synthase [EC 7.1.2.2]) were also detected, though some subunits (corresponding to integral proteins) were missed (as with Rnf) (Fig. 4B). The membrane-bound proteins Nqr and fumarate reductase in our bacteria have also been detected in *Prevotella bryantii* B_1_4^30^, where they have been characterized. Together, these enzymes form a pathway where Rnf is needed to regenerate NAD_red_ and Fd_ox_.

There was one enzyme missing in *P*. *brevis* GA33 and another may be missing in *P*. *ruminicola* 23 (Fig. 4A). In *P*. *brevis* GA33, an enzyme of glycolysis (enolase) was missing in the genome and proteome. This has no easy explanation but has been found previously in the genome^17,31^. In *P*. *ruminicola* 23, an enzyme for converting succinate to propionate, methylmalonyl-CoA decarboxylase (EC 4.1.1._, 7.2.4.3), may also be missing. This enzyme has four subunits, and only three were found in the genome and proteome (MmdA, MmdB, MmdC). The fourth subunit (MmdD) has no defined function^32^, and it may be that the enzyme is still functional with this subunit missing. With a few possible exceptions, our work shows that both *Prevotella* species have the expected enzymes for forming fermentation products in the genome and proteome.

After finding evidence in the genome and proteome, we tested for catalytic activity of the key enzymes (Table 1). We focused mostly on enzymes that generate redox cofactors used by Rnf. Their activities were observed as expected. For example, we found activity of malate dehydrogenase (EC 1.1.1.37), which produces NAD_ox_ (used by Rnf). A similar enzyme producing NADP_ox_ (EC 1.1.1.82) was also detected, but with lower activity. Our work confirms that both *Prevotella* species have the expected enzymes for forming propionate, succinate, and acetate—including those that form NAD_ox_ and Fd_red_.

**Table 1:** Enzyme assays confirm *Prevotella* species catalyze key reactions for forming propionate, succinate, and acetate.

Reaction ID^*a*^	Reaction equation	Source	*P. brevis* GA33		*P. ruminicola* 23	
			Activity^*b*^^,^^*c*^	*P*-value	Activity^*b*^^,^^*c*^	*P*-value
6	D-Glyceraldehyde-3-phosphate + Orthophosphate + NAD^+^ < = > 3-Phospho-D-glyceroyl phosphate + NADH + H^+^	Cell extract	2814 (190)	< 0.001	2840 (360)	0.008
11	2 Oxidized ferredoxin + Pyruvate + CoA < = > 2 Reduced ferredoxin + Acetyl-CoA + CO_2_ + 2 H^+^	Cell extract	346 (97)	0.035	449 (92)	0.020
		Cytoplasmic contents	290 (20)	0.002	291 (84)	0.037
15	Oxaloacetate + NADH + H^+^ < = > (S)-Malate + NAD^+^	Cell extract	979 (30)	< 0.001	2420 (260)	0.006
22	NADH + H^+^ + ubiquinone + n Na^+^[side 1] = NAD^+^ + ubiquinol + n Na^+^[side 2]	Membrane	17.8 (5.6)	0.043	16.4 (2.6)	0.012
		Solubilized membrane	28.8 (1.7)	0.002	17.2 (3.8)	0.022
23	Hydroquinone + Fumarate < = > Quinone + Succinate	Membrane	155.3 (8.9)	0.002	27.5 (5.4)	0.018
		Solubilized membrane	420 (33)	0.003	42.2 (5.9)	0.009
24	ATP + H_2_O + 4 H^+^[side 1] = ADP + phosphate + 4 H^+^[side 2]	Membrane	97 (23)	0.025	53.3 (1.7)	0.001
		Solubilized membrane	102 (16)	0.012	61.5 (4.2)	0.002
25	D-Glyceraldehyde-3-phosphate + Orthophosphate + NADP^+^ < = > 3-Phospho-D-glyceroyl phosphate + NADPH + H^+^	Cell extract	515 (24)	< 0.001	214 (120)	0.106
26	Oxaloacetate + NADPH + H^+^ < = > (S)-Malate + NADP^+^	Cell extract	501.5 (6.7)	< 0.001	216 (25)	0.007

^*a*^See Fig. 4 and Supplementary Tables S3 and S4 for more information.

^*b*^Units are mean (SEM) mU/(mg protein).

^*c*^Results are for at least of 3 biological replicates (cell extract or membrane prepared from independent cultures).

### Possible alternatives to Rnf are not found in Prevotella species

Although Rnf can handle excess NAD_ox_ and Fd_red_ formed during fermentation in these *Prevotella* species, four pathways not involving Rnf can be imagined (see Supplementary Fig. S4A to D). These alternative pathways involve prototypical hydrogenase (EC 1.12.7.2), cytoplasmic ferredoxin:NAD^+^ oxidoreductase (EC 1.18.1.3)^33^, bifurcating hydrogenase (EC 1.12.1.4), and *Campylobacter*-type Nuo^34,35^. Each pathway handles excess NAD_ox_ and Fd_red_ without Rnf. However, none of the appropriate enzymes for these pathways are encoded by the genomes of *P. brevis* GA33 or *P. ruminicola* 23 (Supplementary Tables S3 and S4). Further, we found no H_2_ was formed by *P*. *brevis* GA33 and *P. ruminicola* 23 (Supplementary Fig. S3), ruling out the alternatives involving hydrogenases. 

There are two other, less direct alternatives, and these involve pyruvate dehydrogenase (EC 1.2.4.1) and formate dehydrogenase (EC 1.17.5.3) (see Supplementary Fig. S4E,F). The pathway with pyruvate dehydrogenase, for example, forms no Fd_red_ and is balanced without Rnf. This pathway is in fact the one originally proposed for propionate formation 60 years ago^36^. However, none of the appropriate enzymes are encoded by either genome (Supplementary Tables S3 and S4). Further, we tested for activity of pyruvate dehydrogenase in both *P. brevis* GA33 and *P. ruminicola* 23. We found no activity of this enzyme in either cell extracts or cytoplasmic contents. As a control for this enzyme assay, we tested cell extracts of a bacterium with pyruvate dehydrogenase [*Escherichia coli* BL21(DE3)pLysS], and we found high activity (> 1 U/mg protein). We also found high activity when spiking cell extract of this bacterium into cell extracts of *P. brevis* GA33 and *P. ruminicola* 23. These controls show our assay worked. In sum, there are no obvious alternatives to Rnf in these *Prevotella* species.

### Rnf is found in many organisms forming propionate, succinate, and acetate

We wanted to see if Rnf is distributed widely in organisms that form propionate, succinate, and acetate. To do so, we used phenotypic and genomic data for n = 8,350 prokaryotes from a recent study^37^. The data included fermentative ability (n = 8,350 organisms), fermentation products (n = 1,455 organisms), and genome sequences (n = 4,355 organisms). All organisms were type strains. With this data, we first determined how many organisms form propionate, succinate, and acetate during fermentation. We found that prokaryotes that form exclusively propionate/succinate and acetate (with no other major products) represented about 8.4% of the total. In total, 5.2% of fermentative organisms formed succinate and acetate as major products; 3.0% formed propionate and acetate; and 0.2% formed all three. Thus, fermentations that form propionate, succinate, and acetate were common. 

**Figure 5:**
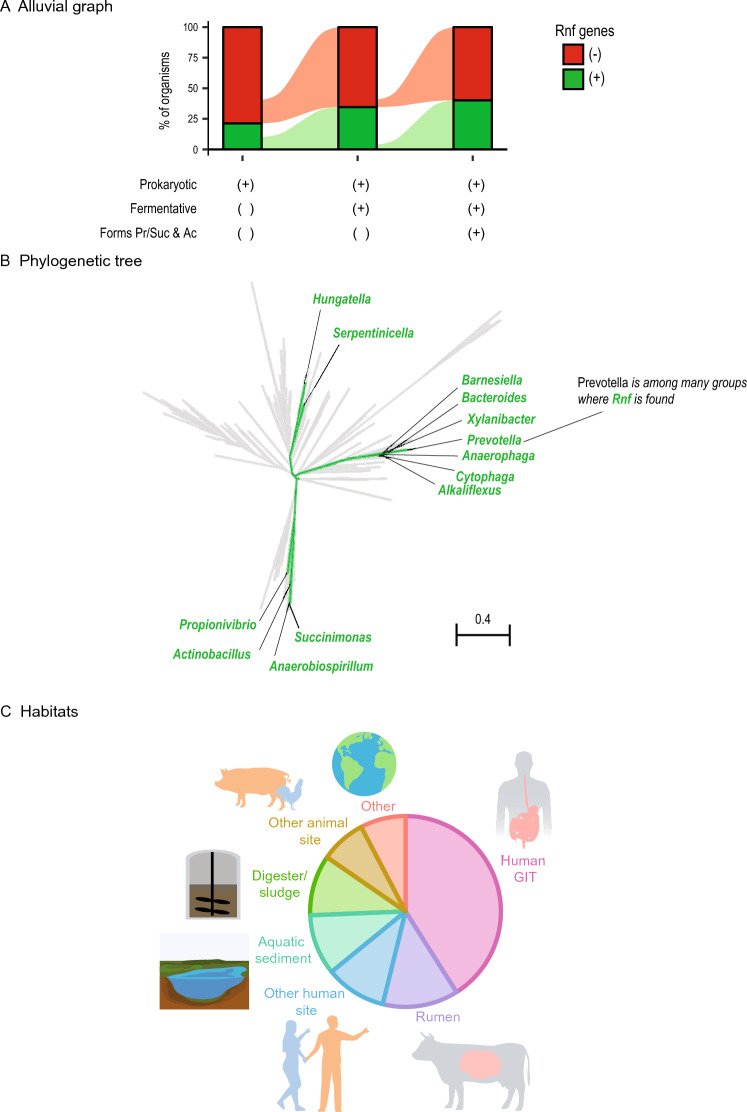
Rnf is found in many prokaryotes that form propionate, succinate, and acetate during fermentation. (**A**) Alluvial graph showing percentage of prokaryotes with Rnf genes. Rnf genes are enriched in organisms that are fermentative and form propionate, succinate, and acetate. (**B**) Phylogenetic tree of prokaryotes, highlighting those with Rnf genes and that form propionate, succinate, and acetate during fermentation. (**C**) Habitats of prokaryotes with Rnf genes and observed to form propionate, succinate, and acetate during fermentation. Abbreviations: Ac, acetate; Suc, succinate; Pr, propionate. See Supplementary Tables S9 and S10 for more information.

Next, we determined the occurrence of *rnf* genes in prokaryotes (Fig. 5, Supplementary Table S9). We found that *rnf* genes were uncommon in prokaryotes in general (Fig. 5A). However, these genes were more common in prokaryotes that are fermentative, and even more so in those that form propionate, succinate, and acetate. This suggests an importance of Rnf in such organisms.

In total, 39 type strains encoded Rnf and formed propionate, succinate, and acetate during fermentation. A phylogenetic tree shows that these strains are diverse, and they belong to several genera (Fig. 5B). Examining their habitats shows that they come from the gut, aquatic sediment, anaerobic digesters, and elsewhere (Fig. 5C, Supplementary Table S10). Together, these results show Rnf is found in many propionate-forming organisms from many habitats.

### Organisms have alternatives to Rnf, but they are uncommon

We used the same genomic and phenotypic data for prokaryotes as before to see if alternatives to Rnf were common in fermentative prokaryotes. We considered the six alternative pathways mentioned above (Supplementary Fig. S4, Supplementary Table S9, Supplementary Table S10). Pyruvate dehydrogenase was the most common, but even still, only 15% of organisms that form propionate, succinate, and acetate encode this enzyme. The four other pathways—involving prototypical hydrogenase, cytoplasmic ferredoxin:NAD^+^ oxidoreductase, bifurcating hydrogenase, formate dehydrogenase, or *Campylobacter*-type Nuo^34,35^—are even less common (Supplementary Fig. S4). 

## Discussion

Our study shows the ferredoxin:NAD^+^ oxidoreductase activity of Rnf is important to forming propionate during fermentation. In two *Prevotella* species, we show that fermentation is apparently unbalanced and produces excess Fd_red_ and NAD_ox_. Rnf handles the excess Fd_red_ and NAD_ox_ by converting them back to Fd_ox_ and NAD_red_ with its ferredoxin:NAD^+^ oxidoreductase activity. No other enzyme (or combination of enzymes) measured had this activity, and several alternatives to Rnf were not found in both *Prevotella* species. Rnf thus is involved in the pathway and allows fermentation to continue.

The pathway for forming propionate has been studied for over 60 years^36^, yet the need for an enzyme like Rnf was only recently recognized^17,18^. A likely reason Rnf has been overlooked is the pathway was first elucidated in propionibacteria^36^. Propionibacteria have pyruvate dehydrogenase, which would make the pathway balanced without Rnf being used (Supplementary Fig. S4)^36^. A pathway with pyruvate dehydrogenase, though plausible, appears seldom used. First, our work shows few organisms forming propionate also encode pyruvate dehydrogenase. Second, propionibacteria themselves may not use this enzyme. Recent work shows they also have pyruvate:ferredoxin oxidoreductase, which is expressed^18,38^ and required for normal growth^18^. In *Prevotella* species, we found activity of pyruvate:ferredoxin oxidoreductase, but not pyruvate dehydrogenase. Thus, there is a real need for an enzyme like Rnf.

Given this need, we looked for direct evidence of Rnf in *Prevotella* species, and we used multiple approaches. Growth experiments showed that redox cofactors would be unbalanced without the ferredoxin:NAD^+^ oxidoreductase activity of Rnf. They showed the problem was serious, and without Rnf, fermentation would halt within 1.5 s. Genomics and proteomics analysis showed that both *Prevotella* species encoded and expressed Rnf. Enzyme assays showed these *Prevotella* species had ferredoxin:NAD^+^ oxidoreductase activity of Rnf, and its properties were as expected. Our experiments also ruled out alternatives to Rnf. For example, one possible alternative is a cytoplasmic ferredoxin:NAD^+^ oxidoreductase. This enzyme was recently characterized in *Clostridium acetobutylicum*^33^, and it is encoded by the butyryl-CoA dehydrogenase operon. However, we did not find genes of this operon in *Prevotella* species, and activity of this enzyme was low or undetectable when directly measured in enzyme assays. Our growth experiments also ruled out H_2_ or formate as other ways of balancing fermentation. Further, our genomics and proteomic analyses do not support involvement of *Campylobacter*-type Nuo. This enzyme oxidizes flavodoxin in *Campylobacter jejuni*^34^, and it has been proposed to oxidize Fd_red_ in *Prevotella copri*^35^. However, our analyses show *Prevotella*, including *P*. *copri*, lack the two subunits that oxidize flavodoxin (NuoF and NuoE). Nonetheless, the presence of the incomplete Nuo complex (NuoABCDHIJKLMN) is intriguing, and it has been observed by others in *Prevotella* species^39^ and other organisms^40^. More experiments are needed to establish the exact function of this complex. In sum, multiple lines of evidence show that *Prevotella* species have Rnf and there is a need for them to balance redox cofactors during fermentation.

Though we considered several alternatives to Rnf, it is not possible to conceive of all possibilities, much less rule them out with approaches used in our work. In this vein, genetic analysis of strains with mutations or deletions of *rnf* genes would be useful. Genetic manipulation systems for *Prevotella* species in this study have not been built due to limited genetic tools available for this group^41^. Recently, great progress has been made in *Prevotella intermedia*^42^, *P. copri*^43^, and other *Prevotella*^44^, which can promote development of new genetic tools for *P. brevis* and *P. ruminicola*. Despite the lack of genetic analysis of Rnf in *Prevotella* species, similar genetic evidence demonstrating that Rnf has ferredoxin:NAD^+^ oxidoreductase activity have been shown in several prokaryotes, such as *Methanosarcina acetivorans*^45^, *B. fragilis*^28^, and *Clostridium thermocellum*^46^. Furthermore, the ferredoxin:NAD^+^ oxidoreductase activity was verified with partially or highly purified Rnf from *Acetobacterium woodii*^47,48^, *Thermotoga maritima*^26^ and *Clostridium tetanomorphum*. Future genetic studies on Rnf in organisms that form propionate, succinate, and acetate can verify its involvement in balancing redox cofactors during fermentation.

Propionate is most commonly formed by succinate pathway, but the acrylate pathway is an alternative. Rnf would be important to either pathway. Though the two pathways involve different carbon intermediates, both produce excess NAD_ox_ and Fd_red_ (2 each per 3 glucose)^17^. Indeed, Rnf is in the proteome of one bacterium that uses the acrylate pathway^49^. During ethanol metabolism via the acrylate pathway in a propionate-producer *Anaerotignum neopropionicum*, Rnf is predicted to operate in the reverse direction to reduce Fd_ox_ and oxidize NAD_red_ at the expense of ATP^50^. Furthermore, Rnf could be important for regenerating NAD_ox_ in strains of *Clostridium saccharoperbutylacetonicum* metabolically engineered to produce propionate via the acrylate pathway^51^. As an aside, one study suggested that *P*. *ruminicola* 23 uses the acrylate pathway, not the succinate pathway^52^. Our study and others^53^ do not support this idea, but Rnf would be important regardless.

The knowledge that Rnf is involved in propionate production is critical for manipulating fermentative propionate production. Modification of metabolic pathways involving redox reactions for synthesis of target metabolites often introduces redox imbalance, which affects the growth and production of the engineered microbes^54^. Several cofactor-engineering strategies have been developed to solve the problematic redox imbalance issue^55,56^; however, it is difficult to address this when the knowledge about enzymes involved in redox balance are unknown.

Our work shows Rnf is encoded by many organisms that form propionate, succinate, and acetate. This result suggests Rnf is important not just to *Prevotella* but many other organisms, although the higher abundance of Rnf in propionate fermenters is not a direct proof that Rnf must be the essential enzyme for redox balance. Our results also show additional strategies exist for balancing redox cofactors. We show five strategies, though none is as common as Rnf. Further, we do not examine eukaryotes, even though they have the same problem^19^—reduced cofactors have to be reoxidized.

In sum, the ferredoxin:NAD^+^ oxidoreductase activity of Rnf is important to the pathway for forming propionate during fermentation. It has importance in the bacteria we study in the rumen and for bacteria from many other habitats. This work is key to understanding how propionate is formed in the environment and to manipulating its production.

## Methods

### Organisms

*P*. *brevis* GA33 and *P*. *ruminicola* 23 were obtained from the ATCC. *C. pasteurianum* 5 and *P. ruminis* A12-1 were obtained from the DSMZ. *S. ruminantium* HD4 was obtained from Michael Flythe (USDA-ARS, Lexington, KY) and originally isolated by Marvin Bryant^57^. *E. coli* BL21(DE3)pLysS was from Promega. 

### Media and growth

Except where noted, strains were grown anaerobically under O_2_-free CO_2_ and with serum bottles with butyl rubber stoppers^58,59^. The inoculant (seed) was 0.1 mL volume of a stationary-phase culture. The temperature of growth was 37 °C. 

*P. brevis* GA33 and *S*. *ruminantium* HD4 were cultured on the medium PC + VFA^58,60^. *P. ruminis* A12-1 was cultured on a complex medium as described by Schoelmerich et al.^29^. *P. ruminicola* 23 was cultured on medium BZ. We developed this defined medium from a complex medium^61^. Per liter, the medium contained 8 g glucose, 0.6 g K_2_HPO_4_, 0.45 g KH_2_PO_4_, 0.45 g (NH_4_)_2_SO_4_, 0.9 g NaCl, 92 mg MgSO_4_, 0.12 g CaCl_2_·2H_2_O, 2 mL of 0.5 g/L hemin in 10 mM NaOH, 1 mL 0.1% (w/v) resazurin, 1 mL SL-9 trace element solution^62^, 10 mL DSMZ-medium-141 Wolin’s vitamin solution, 0.1 mg vitamin B_12_, 322.7 µL isobutyric acid, 322.7 µL 2-methylbutyric acid, 322.7 µL valeric acid, 322.7 µL isovaleric acid, 4 g Na_2_CO_3_, and 1.2 g L-cysteine·HCl·H_2_O. Glucose, Wolin’s vitamin solution, and vitamin B_12_ were added to medium BZ after autoclaving. *C. pasteurianum* 5 was cultured on a glucose medium in 1-L Pyrex bottle sealed with stoppers. Per liter, the medium contained 20 g glucose, 15.329 g K_2_HPO_4_, 1.5 g KH_2_PO_4_, 0.1 g NaCl, 98 mg MgSO_4_, 10 mg Na_2_MoO_4_·2H_2_O, 1 g NH_4_Cl, 50 mg FeSO_4_∙7 H_2_O, 5 mg 4-aminobenzoic acid, and 1 mg biotin. *E. coli* BL21(DE3)pLysS was cultured aerobically on Luria–Bertani medium.

Growth of cultures was measured by removing 1-mL aliquots with a syringe and measuring optical density at 600 nm (OD_600_) in cuvettes in a Thermo Scientific Genesys 20 spectrophotometer. The sample was diluted with 0.9% (w/v) NaCl as needed to remain within the linear range of the instrument.

### Analysis of fermentation products and cells

Three 70-mL cultures were inoculated and grown to the late-log phase (OD_600_ = 1.3 for *P*. *brevis* GA33 and OD_600_ = 4.0 for *P*. *ruminicola* 23). Cells were harvested by centrifugation (21,100 × *g* for 20 min at 4 °C). The supernatant was stored at −20 °C. Cell pellets were resuspended in ddH_2_O and harvested by centrifugation (21,100 × *g* for 30 min at 4 °C). Pellets were transferred to aluminum pans with ddH_2_O and dried at 105 °C overnight. The dry mass of cells was determined by weighing the pan with dried pellet (while still hot)^63^. After cooling to room temperature, the cooled pellet was reweighed to correct for any water absorbed and an aliquot of the cooled pellet was submitted for elemental analysis (C, H, N) by Intertek (Whitehouse, NJ) (Supplementary Table S11).

Supernatant was analyzed for glucose and fermentation products according to Zhang et al.^64^ with modifications. Specifically, acetate was measured by gas chromatography rather than enzymatic assay. Ethanol was measured with a commercial kit from Megazyme (product code K-ETOH).

One aliquot of culture (5-mL) was also collected at the start of the incubation. Cells were removed, and supernatant was analyzed as above. The inoculant for cultures was 0.1 mL of a late-log phase culture. The dry mass of cells in this inoculant was calculated from the volume of inoculant (0.1 mL) and the dry mass of the cells in the late-log phase culture determined by methods above. The elemental composition (C, H, N) was assumed to be the same as cells inoculated and grown to the late-log phase.

### Recovery of carbon and hydrogen

We calculated recovery of carbon in cells and fermentation products. Recovery is defined as the (total carbon at end)/(total carbon at start) × 100%. 

Total carbon (mmol C L^−1^) was the sum of carbon in cells, glucose, fermentation acids, and CO_2_. Carbon in cells (mmol C L^−1^) was calculated from the dry mass of cells (Supplementary Table S12), percent carbon in the dry mass (Supplementary Table S11), and molecular mass of carbon. The dry mass of cells at the start is the dry mass of cells in the inoculant, while the dry mass of cells at the end was directly measured by weighing the dried cell pellet. Carbon in glucose (mmol C L^−1^) was the concentration glucose (Supplementary Table S12) multiplied its carbon number. Similarly, carbon in fermentation acids (mmol C L^−1^) was the summed concentration of each acid (Supplementary Table S12) multiplied by its carbon number. For CO_2_, the concentration at the start was defined as 0. The concentration of CO_2_ at the end was calculated from stoichiometry, assuming −1 CO_2_/formate, 1 CO_2_/acetate, −1 CO_2_/succinate, 2 CO_2_/butyrate, 2 CO_2_/isobutyrate, 1 CO_2_/valerate, 1 CO_2_/isovalerate, and 1 CO_2_/ethanol [after Hackmann et al.^65^]. CO_2_ formed during cell synthesis was ignored.

Recovery of hydrogen was calculated analogously. For H_2_O, we defined the concentration at the start (mmol H L^−1^) as 0. We calculated the concentration at the end (mmol H L^−1^) from stoichiometry, assuming 1 H_2_O/acetate, 1 H_2_O/propionate, and 1 H_2_O/succinate [after Hackmann et al.^65^]. H_2_O formed during cell synthesis was ignored.

### Rate of glucose fermentation

We measured rate of glucose fermentation by *P. ruminicola* 23 in the mid-exponential phase. Samples of culture were collected at 4 points during this phase [where ln(OD_600_) increased linearly over time]. The glucose concentration (mmol L^−1^) was measured as above. The dry cell weight (g dry cell L^−1^) was calculated from OD_600_ (referring to samples where both OD_600_ and weight were known). The rate of glucose consumption [nmol glucose (g dry cell)^−1^ s^−1^] was directly calculated. The rate of glucose fermentation was assumed to be glucose consumption × 0.642 (see Supplementary Table S1). The final value for three biological replicates was 667 nmol glucose fermented (g dry cell)^−1^ s^−1^. 

### Proteomics

We used proteomics to determine what genes were expressed in the cells of *P. brevis* GA33 and *P. ruminicola* 23. Peptide samples from cell extract and cell membrane were prepared and analyzed using LC–MS.

### Preparation of samples for proteomics

For samples of cell extract, proteins were prepared according to Zhang et al.^64^. Briefly, proteins were precipitated by trichloroacetic acid/acetone solution, denatured by urea, reduced by dithiothreitol, alkylated by iodoacetamide, digested by trypsin/Lys-C mix (V5073; Promega), and cleaned with Pierce C_18_ Tips (87784; Thermo Scientific). The eluted peptides were dried by vacuum centrifugation and resuspended in 0.1% (v/v) trifluoroacetic acid.

For the cell membrane, proteins were prepared according to Sievers^66^, with modifications. Cell membrane in the cell extract was pelleted via ultracentrifugation (208,000 × *g* for 60 min at 4 °C; Type 70Ti Rotor and Beckman L8-70 M centrifuge). The pellet was rinsed by gently adding 5 mL Tris-MgSO_4_ buffer and decanting the buffer (with no centrifugation). The pellet was then resuspended in 2-mL Tris-MgSO_4_ buffer (using a pipette to mix), mixed with 15 mL Tris-MgSO_4_ buffer, and pelleted again via ultracentrifugation.

The pellet was resuspended in 2 mL ice-cold carbonate buffer (100 mM Na_2_CO_3_, 100 mM NaCl, pH 11.0) and then 8 mL buffer was added. The resuspended pellet was mixed with a stirrer bar on ice for 60 min. Every 15 min, the sample was further homogenized by drawing it up five times with a syringe and needle. The membrane was harvested by ultracentrifugation, then the pellet was resuspended in 600 μL solubilization buffer (50 mM Tris–Cl [pH 7.5], 8 M urea, 1% (w/v) 3-[(3-cholamidopropyl)dimethylammonio]-1-propanesulfonate). The resuspended pellet was centrifuged (12,000 × *g* for 10 min at 4 °C) and the supernatant was transferred to a 2-mL tube. TCEP solution (500 mM Tris(2-carboxyethyl)phosphine [TCEP] in 200 mM Tris–Cl [pH 8]) was added to 5 mM final concentration. After incubation at 30 °C for 60 min, the sample was alkylated by fresh iodoacetamide (10 mM) at room temperature for 30 min in the dark. The protein concentration was measured with a Pierce BCA protein assay kit (23,227; Thermo Scientific).

The sample was mixed with equal volume of SDS-PAGE sample buffer (125 mM Tris–Cl [pH 6.8], 20% (v/v) glycerol, 20% (w/v) sodium dodecyl sulfate (SDS), a trace of Coomassie Brilliant blue G-250) and loaded into gel wells. After electrophoresis at 140 V for 60 min, the gel (12% acrylamide, 29:1 acrylamide/bis-acrylamide) was fixed in a solution (40% [v/v] ethanol/10% [v/v] acetic acid), washed with distilled water, and visualized by Blue silver stain solution (100 g/L ammonium sulfate, 10% [v/v] phosphoric acid, 1.2 g/L Coomassie Brilliant Blue G-250, 20% [v/v] methanol). The gel lanes loaded with samples were cut into gel pieces. The gel pieces were completely destained with gel washing solution (50% (v/v) acetonitrile LC/MS grade and 50 mM NH_4_HCO_3_ in water), dehydrated by pure acetonitrile, and mixed with diluted trypsin/Lys-C (V5073; Promega) (10 ng/µL) in 50 mM NH_4_HCO_3_ for incubation at 37 °C for 16 h. One µg of trypsin/Lys-C was used for digestion per 8 µg of membrane protein.

Peptides were extracted from the gel pieces by dehydration in acetonitrile, rehydration in 1% (v/v) acetic acid, drying in acetonitrile, 10 min ultrasonication in 5% (v/v) acetic acid, 10 min ultrasonication in acetonitrile, and drying in acetonitrile. The supernatants were pooled, reduced to dryness by vacuum centrifugation, and resuspended in 1% (v/v) trifluoroacetic acid. As for cell extract, the peptides were then cleaned with Pierce C_18_ tips, dried, then resuspended in 0.1% (v/v) trifluoroacetic acid.

### Proteomics using data-dependent acquisition

Peptides were analyzed by LC–MS as described in Zhang et al.^64^. Briefly, the LC was a Dionex UltiMate 3000 RSLC system (Thermo Fisher) equipped with a PepMap C_18_ column (75 μm by 25 cm with a 2-μm pore size; Thermo Scientific). The amount of peptide injected was 1 μg, the flow rate of the mobile phase was 200 μl/min, and the column temperature was 40 °C. The mobile phases were 0.1% formic acid (v/v) in water (A) and 0.1% formic acid (v/v) in acetonitrile (B), and they were used in a gradient elution. The concentration of B was decreased from 10 to 8% over 3 min, increased to 46% over 66 min, increased to 99% over 3 min, held at 99% for 2 min, decreased to 2% over 0.5 min, and held at 2% for 15 min.

The MS was an Orbitrap Fusion Lumos (Thermo Scientific) operated in DDA mode. MS/MS spectra were acquired with automatic gain control target of 5 × 10^3^, ion filling time of 35 ms, and dynamic exclusion time of 50 s with a 10-ppm mass window.

Peptides and proteins were identified from LC–MS/MS data using X!TandemPipeline^67^.

### Proteomics using data-independent acquisition

The peptides were analyzed by LC–MS operating in data-independent acquisition (DIA) mode. The LC was operated as above, except a different gradient elution was used. The concentration of mobile phase B was increased from 2 to 50% over 60 min, increased to 99% over 6 min, held at 99% for 3 min, and decreased to 2% over 2 min.

The MS was the same instrument as above but operated in DIA mode. Mass spectra were acquired using a collision energy of 35%, resolution of 30 000, maximum inject time of 54 ms, and automatic gain control target of 5 × 10^4^. Staggered isolation windows of 12 Da in the range of 400 to 1000 m/z were used.

Data were analyzed with Spectronaut 15 using the directDIA workflow and default settings. Peak area intensities were exported from Spectronaut. Quantitative and statistical analysis was performed, and processing protein peak areas were determined by the Spectronaut software. Prior to library-based analysis of the DIA data, the DIA raw files were converted into htrms files using the htrms converter (Biognosys). MS1 and MS2 data were centroided during conversion, and the other parameters were set to default. The htrms files were analyzed with Spectronaut (version: 15, Biognosys) via directDIA. Precursor and protein identifications were filtered to 1% false discovery rate.

### Enzyme assays

We measured activities of enzymes in cell extract, cell membrane, and cytoplasmic contents. Assays were performed following Zhang et al.^64^. The temperature and other conditions were as reported in Table 2. For assays measuring reduced NAD(P) or ferredoxin, the final component was added after absorbance plateaued. One unit of activity is defined as 1 μmol of product formed per min.

**Table 2:** Conditions used to measure enzymatic activity.

Enzyme	Reference	Assay components^*a*^^,^^*b*^	Product measured (wavelength)	Controls		Conditions^*c*^
Glyceraldehyde-3-phosphate dehydrogenase (EC 1.2.1.12, 1.2.1.13, 1.2.1.59)	After Zheng et al.^68^	50 mM Tricine-Na (pH 8.4), 10 mM potassium phosphate buffer (pH 7), 2 mM dithiothreitol (DTT), 2 mM MgCl_2_, 1 mM glyceraldehyde 3-phosphate, 1 µg of cell extract protein, 1 mM NAD sodium salt or NADP disodium salt	Reduced NAD(P) (340 nm)^*e*^	Cell extract replaced with water		Aerobic
Malate dehydrogenase (EC 1.1.1.37, 1.1.1.82)	After Zeikus et al.^69^	50 mM Tris–Cl (pH 7.6), 0.2 mM NADH disodium salt or NADPH tetrasodium salt, 1 µg of cell extract protein, 2 mM oxaloacetic acid	Reduced NAD(P) (340 nm)^*e*^	Cell extract replaced with water		Aerobic
Pyruvate dehydrogenase (EC 1.2.4.1)	This study	50 mM Tris–Cl (pH 7.6), 10 mM MgCl_2_, 4 mM DTT, 0.2 mM CoA lithium salt, 0.1 mM thiamine pyrophosphate, 4 U/mL phosphotransacetylase, 2 mM NAD sodium salt, 15.1 µg of cell extract or cytoplasmic contents protein, 10 mM sodium pyruvate	Reduced NAD (340 nm)^*e*^	Cell extract or cytoplasmic contents replaced with water		Anaerobic
Pyruvate:ferredoxin oxidoreductase (PFOR) (EC 1.2.7.1, 1.2.7.11)	After Zheng et al.^68^	50 mM Tris–Cl (pH 7.6), 10 mM MgCl_2_, 4 mM DTT, 0.2 mM CoA lithium salt, 30 µM ferredoxin, 0.1 mM thiamine pyrophosphate, 4 U/mL phosphotransacetylase, 15.1 µg of cell extract or cytoplasmic contents protein, 10 mM sodium pyruvate	Reduced ferredoxin (430 nm)^*f*^	Cell extract or cytoplasmic contents replaced with water		Anaerobic
Rnf (Ferredoxin:NAD^+^ oxidoreductase [Na^+^-transporting]) (EC 7.2.1.2)	After Zheng et al.^68^	50 mM Tris–Cl (pH 7.6), 10 mM MgCl_2_, 4 mM DTT, 10 mM NaCl, 80 µg of cell membrane protein (or 40 µg of solubilized cell membrane protein), reduced ferredoxin-regenerating system (0.2 mM CoA lithium salt, 30 µM ferredoxin, 0.1 mM thiamine pyrophosphate, 4 U/mL phosphotransacetylase, 36.2 µg of cytoplasmic contents protein, 10 mM sodium pyruvate), 2 mM NAD sodium salt	Reduced NAD (340 nm)^*e*^	Cell membrane replaced with water		Anaerobic
Nqr (NADH:ubiquinone reductase [Na^+^-transporting]) (EC 7.2.1.1)	This study	100 mM potassium phosphate (pH 6), 100 mM NaCl, 4 mM DTT, 0.4 mM NADH disodium salt, 40 µg of solubilized cell membrane protein or 80 µg of cell membrane protein	Reduced NAD (340 nm)^*e*^	Cell membrane replaced with water		Anaerobic
Fumarate reductase/succinate dehydrogenase (EC 4.2.1.2)	After Asanuma and Hino^70^	100 mM potassium phosphate (pH 6), 100 mM NaCl, 4 mM DTT, 0.4 mM NADH disodium salt, 40 µg of solubilized cell membrane protein or 80 µg of cell membrane protein, 5 mM disodium fumarate	Reduced NAD (340 nm)^*e*^	Fumarate and cell membrane replaced with water		Anaerobic
ATPase (EC 7.1.2.2)	After Schoelmerich et al.^29^	100 mM Tris–Cl (pH 7.4), 5 mM MgCl_2_, 6.25 µg of cell membrane protein or solubilized cell membrane protein, 3.6 mM ATP-DiTris^*d*^	Phosphomolybdate (335 nm)^*g*^	None		Aerobic

^*a*^The components are listed in the order added (with the last component added to initiate the reaction).

^*b*^Source: NADH disodium salt, Sigma N8129; NADPH tetrasodium salt, Calbiochem 481,973; oxaloacetic acid, Sigma O4126; coenzyme A lithium salt, Calbiochem 234,101; ferredoxin, purified from *C. pasteurianum* 5 according to Schönheit et al.^71^, phosphotransacetylase, Megazyme E-PTABS; crude pyruvate:ferredoxin oxidoreductase, cytoplasmic contents from same bacterium being assayed for activity.

^*c*^Anaerobic conditions were 1 mL assay mix in 1.4 mL glass cuvette (Hellma HL114-10–20) capped with chlorobutyl stopper (DWK Life Sciences W224100-081) under N_2_ at 37 °C; aerobic conditions were 0.2 mL assay mix in 96-well plates at room temperature; aerobic conditions for ATPase assay were 0.1 mL assay mix in 1.5 mL tube at 37 °C.

^*d*^Mix was incubated for 0, 4, 8, and 12 min and reaction terminated by adding 14.3 µL of 30% (w/v) trichloroacetic acid.

^*e*^Extinction coefficient of 6,200 M^−1^ cm^−1^, see reference^68^.

^*f*^Extinction coefficient of 13,100 M^−1^ cm^−1^, see reference^72^.

^*g*^Phosphomolybdate was formed by adding 90 µL supernatant with 450 µL of AAM-reagent^73^ and incubating for 10 min at room temperature; phosphate was the standard.

### Preparation of samples for enzyme assays

Samples (cell extract, cell membrane, cytoplasmic contents) were prepared according to methods below. Three 70-mL cultures were grown to mid-exponential phase (OD_600_ = 1.0 to 1.2 for *P*. *brevis* GA33, OD_600_ = 2.7 to 3.0 for *P*. *ruminicola* 23, and OD_600_ = 0.8 to 0.9 for *P. ruminis* A12-1) and then pooled. After growth, culture was transferred to centrifuge tubes. This and all subsequent steps were done anaerobically. All tubes were gassed with O_2_-free N_2_ and sealed with caps containing gas-tight o-rings. 

Cells were harvested by centrifugation (21,100 × *g* for 5 min at 4 °C; F15-8 × 50cy rotor and Sorvall Legend XTR centrifuge). Cells were washed twice with anaerobic Tris-MgSO_4_ buffer (50 mM Tris–Cl [pH 7.6], 20 mM MgSO_4_, 4 mM dithiothreitol, 4 µM resazurin). The washed pellet was resuspended in 20 mL anaerobic Tris-MgSO_4_ buffer. Pierce Universal Nuclease (250 U/µL, Thermo Scientific) was added to a final concentration of 25 U/mL resuspension.

The resuspended cells were lysed with a French press (Glen Mills). The suspension was transferred to a mini-cell pressure cell while gassing with N_2_ and lysed at 110 MPa. Cell debris was removed by centrifugation (14,000 × g for 30 min at 4 °C). An aliquot (c. 2 mL) of the cell extract was stored under N_2_ at −80 °C.

Cell membrane and cytoplasmic contents was prepared from cell extract. Using the remaining cell extract (c. 17 mL), membranes were harvested by ultracentrifugation (208,000 × *g* for 60 min at 4 °C; Type 70Ti Rotor and Beckman L8-70 M centrifuge). The supernatant (cytoplasmic contents) was stored under N_2_ at −80 °C. The pellet was rinsed by gently adding 5 mL anaerobic Tris-MgSO_4_ buffer and decanting the buffer (without centrifugation). It was then resuspended in 2 mL anaerobic Tris-MgSO_4_ buffer, using a pipette to mix. The resuspended pellet (cell membrane) was stored under N_2_ at −80 °C.

Solubilized cell membrane was prepared similarly to cell membrane, with a few modifications. The cell pellet was resuspended in 21 mL anaerobic Tris-sucrose buffer (50 mM Tris–Cl [pH 8.0], 20% [w/v] sucrose, 4 mM dithiothreitol, and 0.4 µM resazurin). The resuspended pellet was first treated by adding lysozyme (0.1 mg/mL) and then by adding ten volumes of 0.1 M sodium EDTA (pH 7.0). The resuspended pellet was incubated at 37 °C for 15 min after each addition. The treated pellet was harvested by centrifugation (21,100 × *g* for 15 min at 4 °C). It was resuspended in 20 mL anaerobic Tris-FMN buffer (50 mM Tris–Cl [pH 7.6], 5 µM riboflavin 5’-monophosphate sodium salt hydrate [FMN, Sigma F6750], 20 mM MgSO_4_, 4 mM dithiothreitol, 4 µM resazurin), mixed with 2 µL of Pierce Universal Nuclease (250 U/µL). Cells were lysed with the French press and membranes were harvested by ultracentrifugation as above. Membranes were rinsed with 5 mL anaerobic Tris-FMN buffer and resuspended in 2.5 mL of the same buffer. An aliquot (c. 2.3 mL) was incubated with n-dodecyl β-D-maltoside (DDM; 1 mg/mg cell protein) on ice with a stir bar inside to mix for 120 min. Undissolved membranes were removed by ultracentrifugation (208,000 × *g* for 60 min at 4 °C), and the supernatant stored under N_2_ at −80 °C.

All fractions (cell extract, cytoplasmic contents, cell membrane, solubilized cell membrane) were prepared at least three times per strain.

The cell extract of the *E. coli* strains was prepared in the same way as those for *Prevotella* species but was done aerobically. The cells grown overnight in 200 mL LB medium was collected by centrifugation. The buffer used to wash and resuspend cells contained 50 mM Tris–Cl [pH 7.2] and 10 mM MgCl_2_.

### Preparation of ferredoxin

When required in assays, ferredoxin was purified from *C. pasteurianum* 5 was according to reference^71^ with modifications as indicated below. A 900-mL culture of *C. pasteurianum* 5 was grown to late log phase (OD_600_ = 4.0 to 6.0). Cells were harvested by centrifugation (12,000 × *g* for 10 min at 4 °C; F15-8 × 50cy rotor and Sorvall Legend XTR centrifuge). Cells were washed once with 15 mL of 50 mM Tris–Cl (pH 7.2), 10 mM MgCl_2_, and the cell pellet was stored at −20 °C.

Ferredoxin was purified from cells according to Schönheit et al.^71^, with modifications. Cells were lysed by resuspending in 1.5 mL dH_2_O/g cell pellet, adding 500 µg lysozyme and 25 U Pierce Universal Nuclease (Thermo Scientific 88,700)/mL resuspended cells, then incubating at 37 °C for 40 min. The lysate was adjusted to pH 6.8 with 1 M Tris-base, and conductivity was adjusted to that of 100 mM NaCl using 2 M NaCl. Non-ferredoxin proteins were precipitated by adding 1.2 g of cold (−80 °C) acetone/g lysate. These proteins were removed by centrifugation (24,400 × *g* for 10 min at 4 °C).

Ferredoxin was precipitated with 50 µL of 10% (w/v) polymin-P (BASF 50,019,138)/mL acetone supernatant, centrifuged, and resuspended in 1 mL ammonium sulfate (60% saturated, pH 6.8)/g original cell pellet. The resuspended ferredoxin was centrifuged again, and the pellet was discarded.

Ferredoxin was further purified with DEAE-cellulose (Santa Cruz Biotechnology sc-211213). It was bound to DEAE-cellulose resin by mixing with 30 mg resin/mL cell pellet for 2 to 4 h. The DEAE-cellulose was centrifuged, washed by adding 0.5 to 1 mL ammonium sulfate (2:1 mixture of 60% saturated [pH 6.8] and 90% saturated [pH 6.8] ammonium sulfate)/g cell pellet, then centrifuged again. The wash step was repeated. Ferredoxin was eluted from DEAE-cellulose with a small volume of ammonium sulfate (10% saturated, pH 6.8) and vortexing. The DEAE-cellulose was removed by centrifuging (21,100 × *g*, 15 min at 4 °C; Thermo Sorvall Legend Micro 21R). Purity was measured according to the ratio of absorbance at 390 and 280 nm (A_390_/A_280_). Ferredoxin with A_390_/A_280_ ranging from 0.7 to 0.75 was concentrated via ultrafiltration using a filter with molecular weight cutoff of 3 kDa (Fisher 88,525).

The final concentration of ferredoxin was 900 to 1000 µM with a yield of 8 mg per 80 g of cells. The yield was estimated using an extinction coefficient at 390 nm of 30/mM/cm. The ferredoxin was stored at −80 °C until use.

### Other chemical analyses

Protein was measured using the Bradford method^74^. The standard was bovine serum albumin.

H_2_ gas was measured using gas chromatography. The gas chromatograph was a Trace 1300 equipped with a thermal conductivity detector (TCD) (Thermo Scientific). The column was a TG-BOND Q (30 m × 0.53 mm i.d. coated with 20 µm film thickness; Thermo Scientific). N_2_ (2.5 mL/min flow rate) was the carrier gas. Headspace samples from cultures (1 mL) were manually injected with a gas tight syringe (Hamilton #81356). Injection was performed in split mode, with the split flow rate 75 mL/min, split ratio to be 30. The purge flow rate 3.0 mL/min. The back inlet had a temperature of 150 °C. The initial oven temperature was 40 °C, maintained for 3 min, raised to 150 °C at 25 °C/min, and finally held at 150 °C for 2 min. The TCD had a temperature of 150 °C, the filament temperature was 200 °C, and the reference gas flow rate was 1.5 mL/min. Data handling was carried out with Chromeleon Chromatography Data System software (Thermo Scientific).

### Searches for genes and proteins

We searched genomes for genes involved in forming propionate, succinate, and acetate. To do so, we used IMG/M database^75^, the IMG/M genome ID for each genome, and the KEGG Orthology (KO) ID^76^ for each gene. For some genes, we searched for the COG^77^ or pfam^78^ ID instead. We classified genes for hydrogenases using HydDB^79^ according to Hackmann^37^.

For each gene, we report the respective enzyme name, enzyme symbol, EC number, and biochemical reaction. This information came from KEGG^76^ and HydDB^79^. An enzyme was considered present in the genome if genes for all subunits was found. A reaction was considered present if at least one isozyme was found.

### Other bioinformatic analyses

Proteomes were searched for proteins using locus tags for genes above. Phylogenetic trees were constructed according to Hackmann and Zhang^80^. We identified habitats of organisms forming propionate, succinate, and acetate using *Bergey’s Manual*^81^, BacDive^82^, and information from public culture collections. 

### Statistics

A one-sided *t*-tests was used to determine if mean yield of fermentation products and mean values of enzymatic activity was greater than 0. *P*-values reported are for that test. 

### Supplementary Information

Supplementary Information 1.

Supplementary Information 2.

## Data Availability

The LC–MS datasets generated for this study can be found in the Proteomics Identification (PRIDE) Archive with the dataset identifier PXD034119. Other datasets used and/or analyzed during the current study available from the corresponding author on reasonable request.
