# Supplementary material for: The oxidoreductase activity of Rnf balances redox cofactors during fermentation of glucose to propionate in Prevotella
Source: Sci Rep. 2023 Sep 30;13:16429. doi: 10.1038/s41598-023-43282-9 (PMC10542786; doi:10.1038/s41598-023-43282-9)
Supplement: Supplementary file 1 — Supplementary Information 1. [file 41598_2023_43282_MOESM1_ESM.pdf]

Supplementary Figures

The oxidoreductase activity of Rnf balances redox cofactors during fermentation of glucose to propionate in *Prevotella*

Bo Zhang[1], Christopher Lingga[1], Hannah De Groot[1], Timothy J. Hackmann[1]✉

[1]Department of Animal Science, University of California, Davis, CA, USA

✉Email: tjhackmann@ucdavis.edu

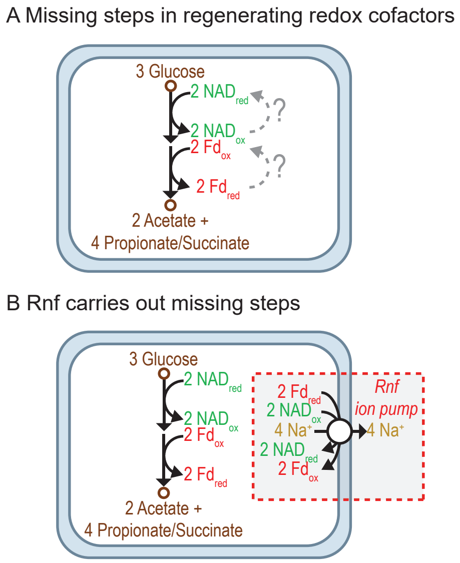

Fig. S1. Fermentation of glucose to propionate, succinate, and acetate has missing or unknown steps. (A) The missing steps are for regenerating redox cofactors. (B) We hypothesize Rnf carries out the missing steps. Abbreviations: Fd_{ox}, oxidized ferredoxin; Fd_{red}, reduced ferredoxin (two reduced iron-sulfur clusters); NAD_{ox}, oxidized NAD; NAD_{red}, reduced NAD.

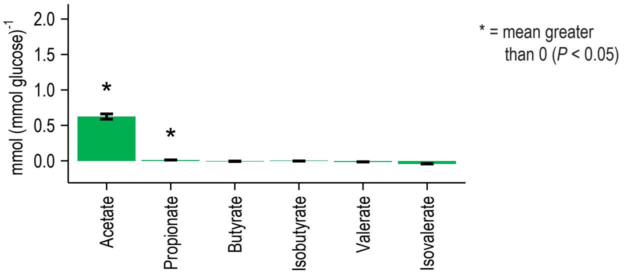

Fig. S2. *Prevotella brevis* GA33 forms trace amounts of propionate. Experiments are as in Fig. 1, except propionate was withheld from the medium (PC + VFA). Propionate is in high concentrations in normal media (c. 6.4 mmol L^{-1}), and withholding it allowed propionate formation to be detected more sensitively. Products not shown were not measured.

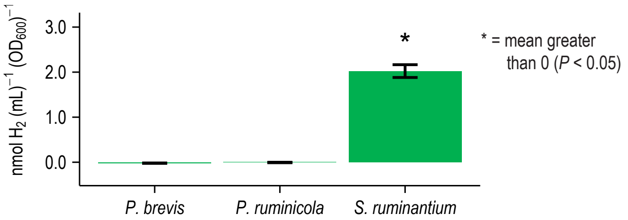

Fig. S3. *Prevotella* do not form H_2 during fermentation of glucose. *Selenomonas ruminantium* HD4 is known to form trace amounts of H_2 and is included as a control.

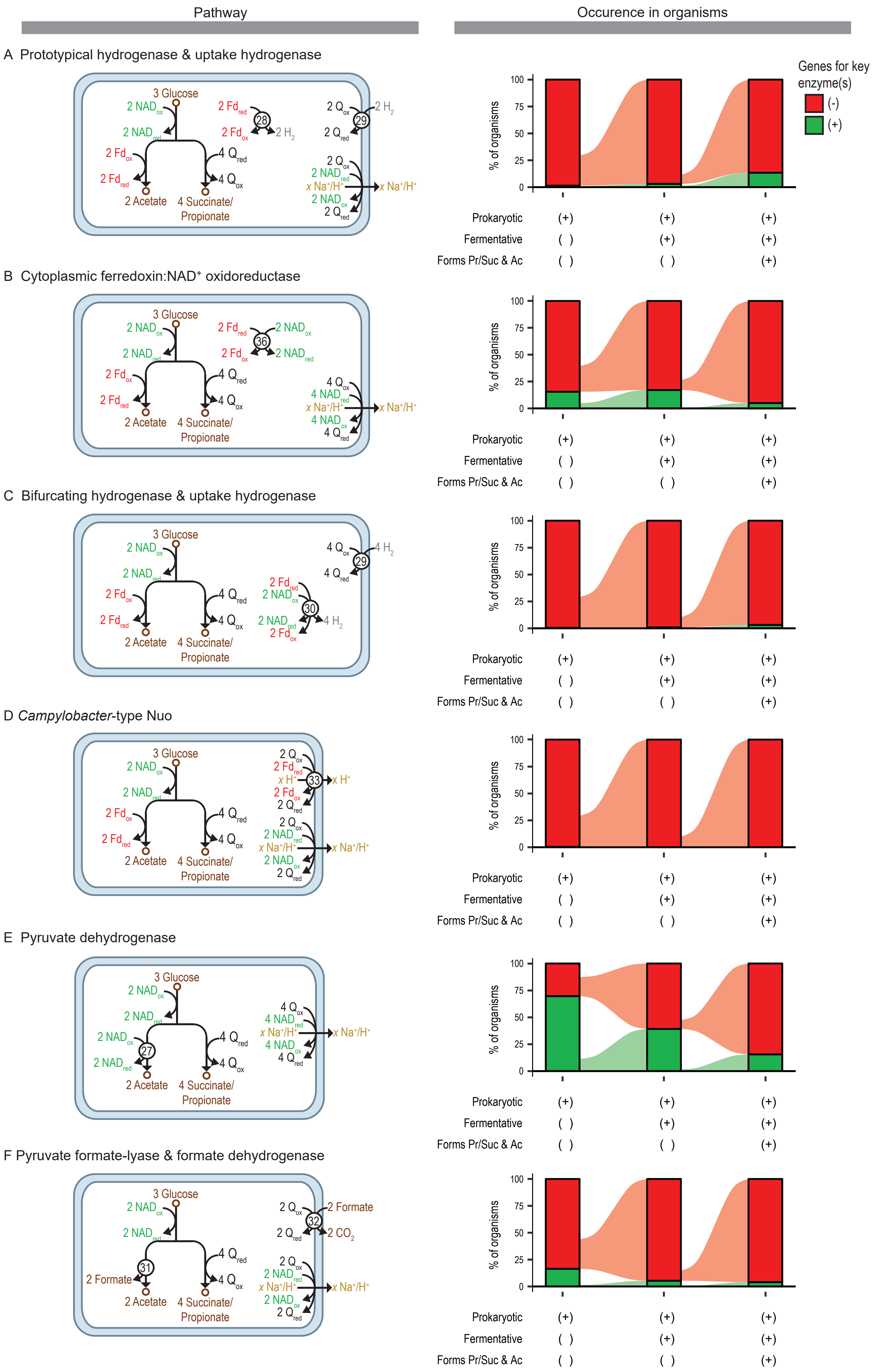

Fig. S4. Rnf is not needed in all pathways that form propionate, succinate, and acetate, but these alternatives are uncommon. Alternatives to Rnf involve (A) prototypical hydrogenase and uptake hydroge-nase, (B) cytoplasmic ferredoxin:NAD$^+$ oxidoreductase, (C) bifurcating hydrogenase and uptake hydrogenase, (D) *Campylobacter*-type Nuo, (E) pyruvate dehydrogenase, and (F) pyruvate formate-lyase and formate dehydrogenase. Conversion of Q_{red} to Q_{ox} is drawn in middle of cell, but it actually occurs at membrane. Abbreviations: Fd_{ox}, oxidized ferredoxin; Fd_{red}, reduced ferredoxin (two reduced iron-sulfur clusters); NAD_{ox}, oxidized NAD; NAD_{red}, reduced NAD; Q_{ox}, oxidized quinone; Q_{red}, reduced quinone.
